# Primary Ciliary Dyskinesia and Type 1 Diabetes: True Association or Circumstantial?

**DOI:** 10.7759/cureus.39344

**Published:** 2023-05-22

**Authors:** Sarrah M Fadl, Mustafa Kafaji, Hesham Abdalla, Maryam A Dabbour, Abdullah Al-Shamrani

**Affiliations:** 1 Pediatric Pulmonology, Prince Sultan Military Medical City, Riyadh, SAU; 2 General Surgery, College of Medicine, Alfaisal University, Riyadh, SAU; 3 Medicine, Alfaisal University, Riyadh, SAU

**Keywords:** insulin resistance, mutation, genetic testing, respiratory tract infections, diabetes mellitus

## Abstract

Primary ciliary dyskinesia (PCD) is a rare autosomal recessive inherited heterogeneous respiratory disorder. The diagnosis of PCD is challenging and necessitates a multi-test diagnostic approach because there are no gold standard diagnostic tests available to confirm PCD. However, rapid advancement in understanding the molecular genetic basis of PCD has greatly improved PCD diagnosis. Studies have reported that PCD may increase the risk of rheumatoid arthritis, congenital heart disease, severe esophageal diseases, and others. Therefore, the present study aimed to assess the risk of type 1 diabetes mellitus (T1DM) in a genetically confirmed PCD patient. In this case study, an 11-year-old girl with autosinopulmonary infections and her younger brother were diagnosed with PCD. The patient’s DNA was extracted for next-generation exome sequencing. Our analysis of the exome sequencing data revealed the PCD-causing genetic variant p.Glu286del in the RSPH9 gene on chromosome 6p21.1. In addition, the biochemical findings at the time of patient’s admission showed elevated glutamic acid decarboxylase antibodies, HbA1c, and ketone levels, with impaired glucose tolerance, which indicated the presence of T1DM. In conclusion, the clinical features, biochemical reports, and genetic testing confirmed PCD in this patient and the possible association between PCD and T1DM.

## Introduction

Primary ciliary dyskinesia (PCD) is a genetically heterogeneous autosomal recessive disorder characterized by abnormalities in the structure and function of cilia in different body organs such as respiratory system, ears, nose, throat, heart, genital system, etc. due to mutations in RSPH9 and RSPH4A genes. The global prevalence of PCD is estimated to be between 1:20,000 and 1:40,000 live births [1,2]. Primary ciliary dyskinesia is a challenging and multifaceted disorder with unknown pathophysiology and a complicated diagnosis [3]. Several diagnostic approaches are currently in use, including the measurement of nasal nitric oxide, electron microscopy, high-speed video microscopy, immunofluorescent staining, etc. to help with the diagnosis of PCD. However, none of them can be considered the gold standard because of their low accuracy and inconsistent results. Therefore, diagnosis requires a number of technically demanding, sophisticated investigations [4,5].

Recent advances in genetic approaches, such as the use of next-generation sequencing technologies, have enabled the discovery of an increasing number of new variants associated with PCD [1]. Approximately 70% of people with PCD are currently diagnosed using commercial panels due to mutations in known PCD genes [6]. To date, more than 50 PCD-associated mutated genes have been identified and found to be involved in ciliary biogenesis, assembly, structure, and function. Therefore, these variants could be used as biomarkers for the early identification and treatment of PCD.

A previous study reported that genetic, immunologic, and environmental factors may increase the risk for type 1 diabetes mellitus (T1DM) [7,8]. The role of PCD in the development of T1DM has yet to be determined. However, complex congenital heart disease and severe esophageal disease have been linked to PCD [9]. The coexistence of Kartagener's syndrome and rheumatoid arthritis has also been reported [10]. Research evidence supports a probable association between PCD and T1DM in both human and animal subjects [11]. Hence, the present study aimed to assess the risk of T1DM in a genetically confirmed PCD patient.

## Case presentation

An 11-year-old girl was admitted on June 22, 2021, to the pediatric emergency department of Prince Sultan Military Medical City (PSMMC) for a period of three days with a productive cough, which was not responding to bronchodilator, and shortness of breath. The recruitment for the present case was performed in accordance with the CARE (for CAse REports) checklist. The principles of the Declaration of Helsinki were followed to complete this research study. The patient's history revealed a one-month pediatric intensive care unit (PICU) stay due to severe respiratory syncytial virus bronchiolitis. There were no further hospitalizations until she was six years old, when she presented with a history of weight loss to the 25th centile (previously in the 50th centile), according to reference growth charts [12]. Additionally, a history of polyuria, decreased oral intake, and burning micturition were reported.

The biochemical findings at the time of patient admission showed elevated levels of glutamic acid decarboxylase (GAD) antibodies, HbA1c, ketones, and glucose. The microbiological examination showed *Escherichia coli* cystitis. Based on these clinical findings, the patient was diagnosed with T1DM, and insulin was started. One year later, at the age of seven, she presented with earache and bilaterally reduced hearing, as well as symptoms of upper respiratory tract infection, and was treated with antibiotics for bilateral otitis media with effusion. The symptoms worsened, so bilateral ventilation tube insertion and an adenoidectomy were performed. At the same time, her brother was diagnosed with an autosomal recessive pathogenic variant in the RSPH9 gene confirming her brother's PCD while parents were carriers. This justified a targeted genetic test for the present case. It indeed validated the presence of the same mutation, and a PCD diagnosis was made. Parents were consanguineous, yet neither had developed PCD.

Physical examination

We found normal vital signs, grade 1 clubbing, an unremarkable chest examination, yellowish-green sputum output, and no evidence of purulent nasal discharge. In the audiogram, type B tympanograms were also detected bilaterally. The results of other systemic examinations were normal, and the patient's 50th percentile was reported as 32 kg. All procedures performed in this study were in accordance with the ethical standards of the institutional and/or national research committee(s).

Investigations

Laboratory Findings

The biochemical analysis showed the levels of C-reactive protein (CRP), procalcitonin, HbA1C, and glutamate decarboxylase (IU/mL) were comparatively higher in our case as compared to reference values. Other values, such as WBC, neutrophils, lymphocytes, hemoglobin, platelets, thyroglobulin antibodies, and thyroid peroxidase antibodies, were within the normal range. In our hospital, the insulin secretion capacity test was not available; therefore, we could not perform it. Sputum culture revealed squamous epithelial cells and moderate white blood cells (Table 1). However, the blood culture reports were negative, while the urine culture was positive for proteus mirabilis. The fractional exhaled nitric oxide (FeNO) level was 49 parts per billion, which affects the level of inflammation.

**Table 1:** Results for various biochemical parameters estimated WBC = white blood cell, GAD = glutamic acid decarboxylase, CRP = C-reactive protein, DM = diabetes mellitus

Biochemical parameters	Results	Reference
WBCs	6.9 × 10^9^/L	4.5-11.0 × 10^9^/L
Neutrophils (%)	51	38.7%-76.7%
Lymphocytes (%)	39	10.0%-47.0%
Hb (g/dL)	14	11.5-15.5 g/dL
Platelets	393 × 10^9^/L	150-450 × 10^9^/L
CRP (mg/L)	4.33	0.0-6.0 mg/L
Procalcitonin (ng/mL)	0.23	<0.05 mic/L
HbA1C (%)	8.6 and 12.8	>8, action suggested for DM; <7, aim for diabetic control; <5.7, non-diabetic level; 5.7-6.4, pre-diabetic; ≥6.5, diabetic
Thyroglobulin antibody (IU/mL)	10	0.0-115 IU/L
Thyroid peroxidase (IU/mL)	9	0.0-34 IU/L
Glutamate decarboxylase (IU/mL)	93	0.0-5 IU/mL
Anti-IA (IU/mL)	2	0-10 IU/mL
GAD (nmol/L)	0.02	<0.02 nmol/L

Radiological Findings

**Figure 1:**
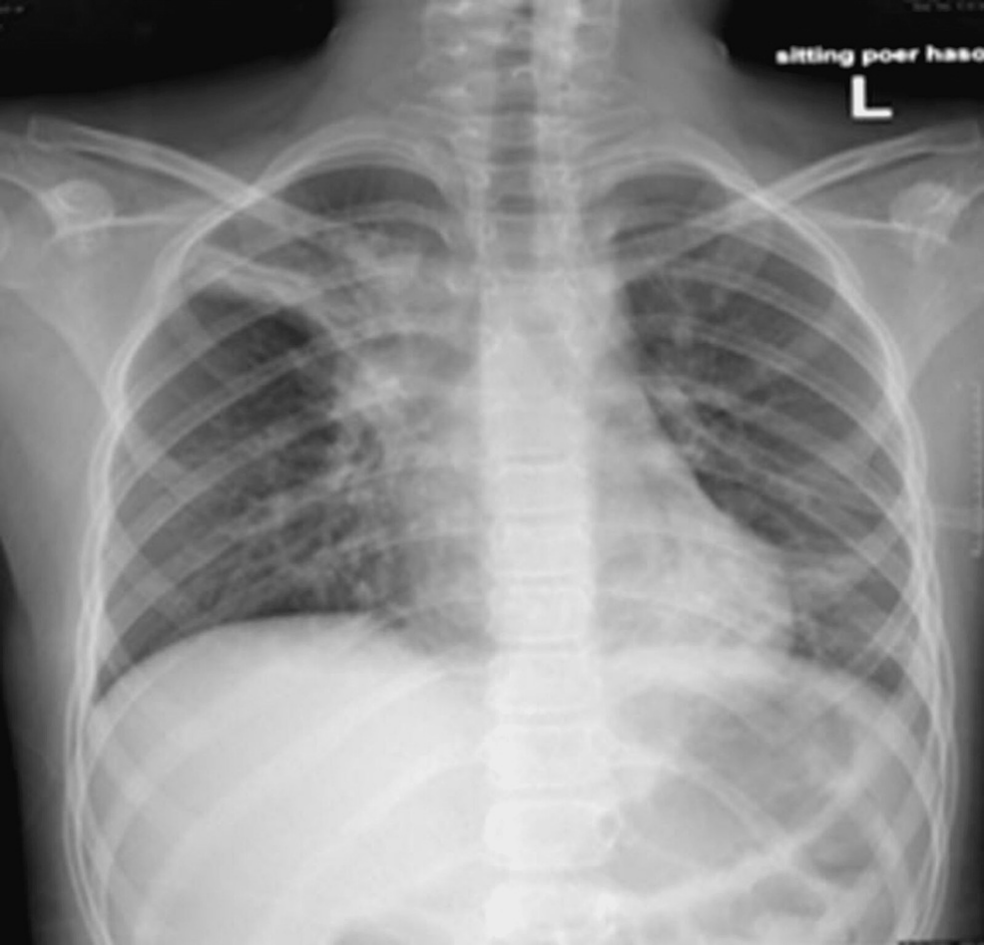
Chest X-ray showing bilateral peribranchial pneumonic infiltration, especially in the right upper zone, and subsegmental lung atelectasis

Her chest X-ray showed bilateral peribranchial pneumonic infiltration, especially in the right upper zone, and subsegmental lung atelectasis (Figure 1).

**Figure 2:**
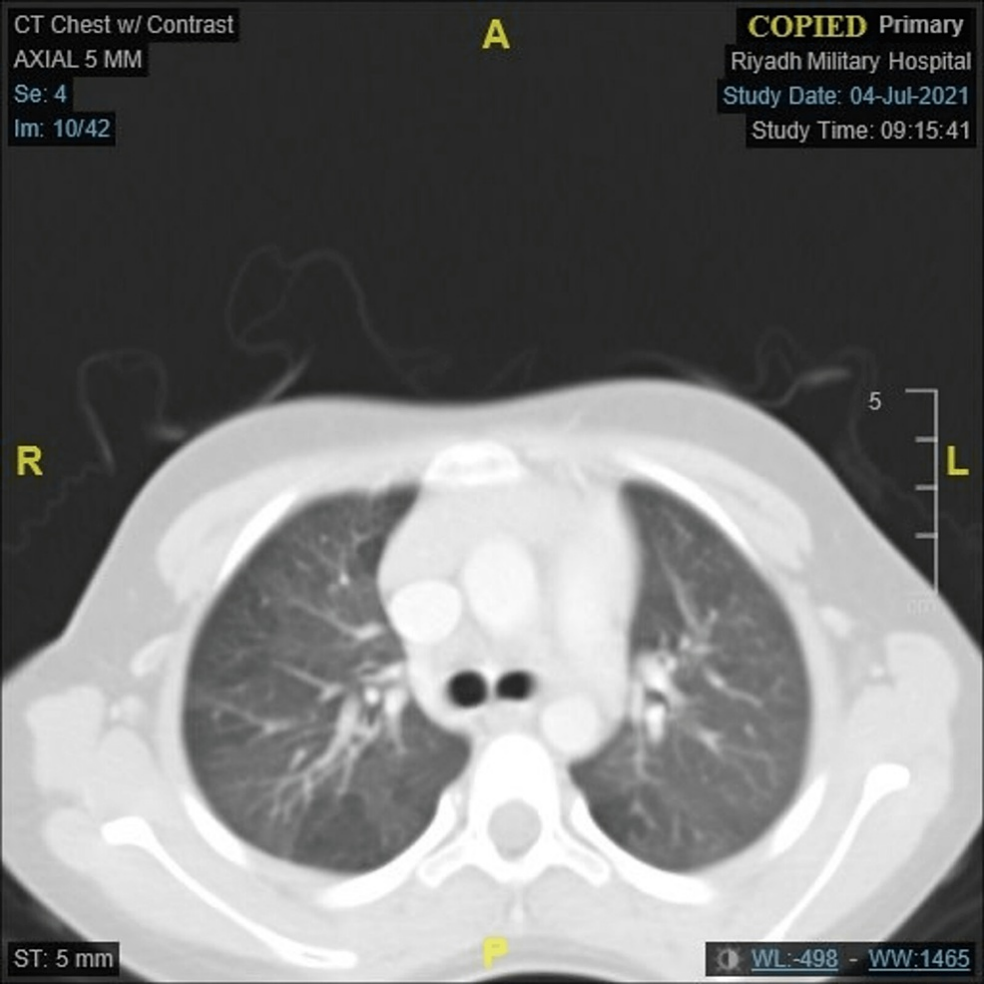
A CT scan showing the axial lung window, diffuse mosaic, ground-glass attenuation of the lungs suggestive of regional air trapping with no evidence of bronchiectasis

When a CT scan of the chest was performed, it indicated diffuse mosaic, ground-glass attenuation of the lungs, suggesting regional air trapping (Figure 2).

**Figure 3:**
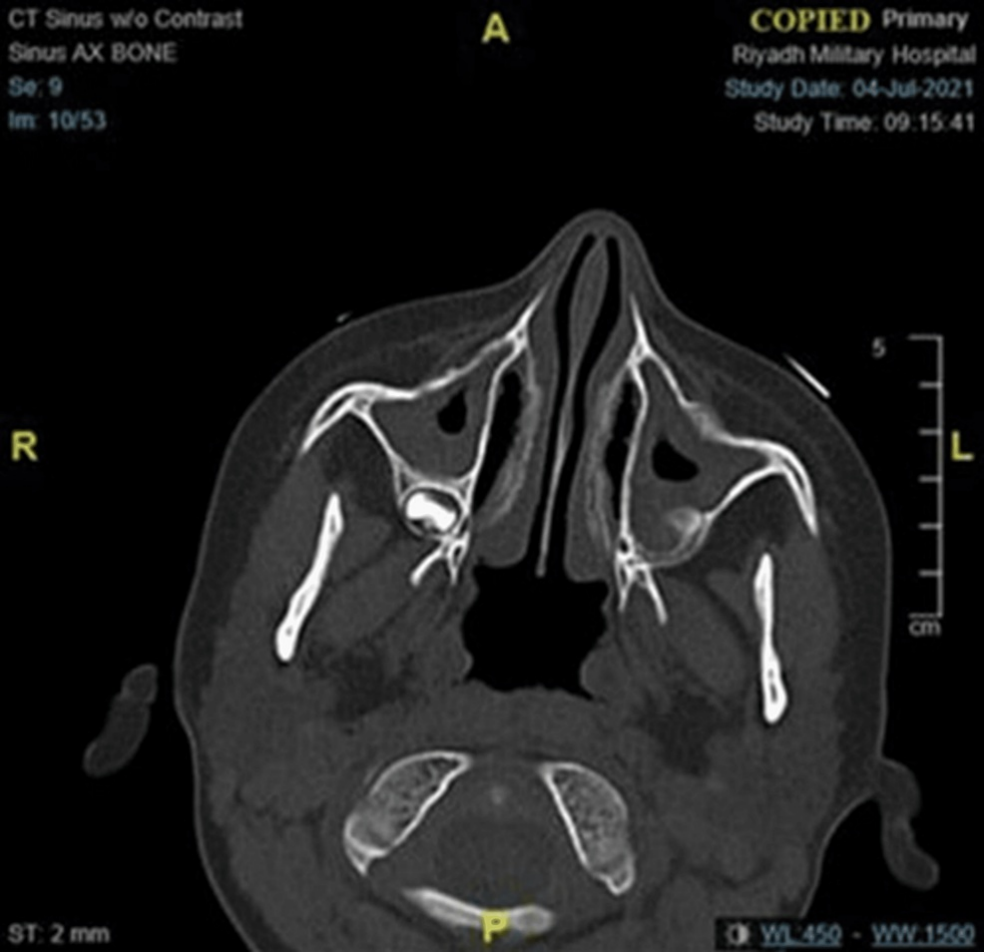
Sinus CT consistent with mucosal thickening of small sphenoid sinuses, ethmoid and maxillary sinuses; nasal septum is intact and middle ear cavities are well aerated

Furthermore, a CT scan of the sinuses revealed mucosal thickening in the small sphenoid, ethmoid, and maxillary sinuses (Figure 3).

Management

The patient was administered IV fluids and IV tazocin for two weeks. We cleared her airways with a 7% saline solution and salbutamol and performed chest physical therapy every eight hours. Dornase alfa nebulization was commenced prior to chest physiotherapy that showed a good response.

On admission, her blood sugar level was at its highest (133 mg/dL), but was later stabilized with her regular dose of 18 IU of insulin. There were no adverse events noticed while the patient was being managed. Upon recovery, additional PCD education and family planning services were offered to increase awareness among the parents.

## Discussion

Despite substantial advances in the clinical understanding of PCD, it is still a diagnosis that is not often suspected, which can result in a delay in diagnosis regardless of early lifetime symptoms [13]. Determining associations between PCD and other well-established diseases leads to a better understanding of the disease's pathophysiology, its early detection, and appropriate therapeutic intervention. This can help decrease the use of expensive and time-consuming genetic investigations and offer better treatment and management plans. This case study highlights an interesting potential association between T1DM and PCD for the first time.

There are several reasons why PCD in this case study did not manifest until the patient was six years old. First, she was the firstborn of consanguineous parents with little to no experience or education on respiratory symptoms and their importance. Another is her repeated, untreated sinopulmonary infection, which resulted in serious lung damage and necessitated the placement of bilateral breathing tubes [14]. The patient’s final diagnosis was eventually revealed after we determined that her younger brother was diagnosed with PCD at the age of two years, presenting with earlier and more acute symptoms; however, her brother had normal glucose levels. Because T1DM usually appears in children at four years of age or older and her two-year-old brother’s glucose levels were normal, he received no further diabetic tolerance tests.

Despite the fact that there are currently no gold standard diagnostic techniques for PCD, we decided to genotype this patient due to the high suspicion and positive family history [15]. In our patient, the exome sequencing data revealed the presence of the PCD-causing genetic mutation p.Glu286del in the RSPH9 gene on chromosome 6p21.1. This gene encodes a protein that is considered to be a part of the radial spoke head in motile cilia and flagella. Studies conducted in Saudi Arabia reported that the prevalence of RSPH9 gene mutations is 31% in PCD, which demonstrates a substantial association between RSPH9 gene mutations and PCD in the Saudi population [16]. In previous studies, an association between rheumatoid arthritis and Kartagener's syndrome was reported. These studies identified B27, DR4, and DR1 as common genes in patients with both disorders [17,18].

The impact of cilia on glucose metabolism and pancreatic functions has been reported previously. Hughes et al. investigated the extremely crucial role that cilia play in regulating both pancreatic B-cell function and energy metabolism [19]. To describe the role of cilia in pancreatic B-cell and islet function, they used genetically modified mice with deleted pancreatic B-cell genes that cause consequential B-cell secretory failure in addition to aberrant α- and δ-cell hormone secretion and altered systemic energy metabolism. These genetically modified mice have also shown hypo-insulinemia and an abnormal glycemic state. These findings suggest that cilia not only play a significant role in intrinsic B-cell function but also mediate communication between islet cells and other metabolic tissues. To understand the associations between T2DM and ciliary dysfunction, a decrease in glucose-stimulated first-phase insulin secretion in the β-cell line of pancreatic islets in mice was observed [17]. Furthermore, increased oxidative stress in diabetic patients may also damage respiratory cilia, resulting in impaired ciliary clearance and subsequent ciliary dysfunction [20]. Shorter et al. reported an association between cilia-specific autoantibodies and T1DM while studying the structural alterations of oviductal cilia in female diabetic mice [21]. The biochemical findings in our case study revealed high glutamate decarboxylase levels as compared to the normal rage. A high glutamate decarboxylase level has been associated with increased oxidative stress [22]. Oxidative stress can impair ciliary function by increasing leukocytes and inflammation, and damage the DNA of genes linked to PCD.

## Conclusions

A multidisciplinary disease management approach is required to limit the progression of PCD because it is a multifactorial, complex chronic disorder. The possible association of PCD with T1DM may cause compromised immunity and increased vulnerability to infection, which could have a negative impact on PCD patients. Since both T1DM and PCD have a strong genetic background, further genetic studies may assist in finding novel evidence of an association between these two diseases. This could lead to a better understanding of the pathophysiology of PCD, appropriate screening of high-risk patients at an early stage of the disease, and the development of new diagnostic markers and treatment strategies. We recommend the future genome-wide association studies to find out the frequency of this novel variant and then targeted sequencing for the early screening of high-risk patients. Further studies are required to validate and strengthen the present findings.
